# Serum Biomarkers in Acute Ischemic Stroke: Clinical Applications and Emerging Insights

**DOI:** 10.3390/jcm14217748

**Published:** 2025-10-31

**Authors:** Anthi Tsogka, John Ellul, Elisabeth Chroni, Apostolos Safouris, Klearchos Psychogios, Dimitra Veltsista, Odysseas Kargiotis

**Affiliations:** 1 Department of Neurology, University Hospital of Patras, School of Medicine, University of Patras, 26504 Patras, Greece; tsogka.anthi@gmail.com (A.T.); ellul@upatras.gr (J.E.); echroni@yahoo.com (E.C.); dveltsista@yahoo.gr (D.V.); 2 Stroke Unit, Metropolitan Hospital, 18547 Piraeus, Greece; safouris@yahoo.com (A.S.); apsychoyio@yahoo.gr (K.P.); 3 Second Department of Neurology, “Attikon” University Hospital, School of Medicine, National & Kapodistrian University of Athens, 12462 Athens, Greece

**Keywords:** acute ischemic stroke, serum biomarkers, uric acid, D dimer, fibrinogen, troponin, Pro BNP, C-RP, Inteleukin-6, GFAP, NFL

## Abstract

Acute ischemic stroke (AIS) remains a major cause of long-term disability and death worldwide, posing significant challenges to healthcare systems. Timely diagnosis is crucial, as acute phase therapeutic options are highly time-sensitive and most effective when administered early in the disease course. In this context, serum biomarkers have emerged as a promising and complementary tool to aid in the rapid and accurate diagnosis, prognosis, and therapeutic monitoring of AIS. This narrative review aims to provide a comprehensive overview of the current landscape of serum biomarkers relevant to AIS. These biomarkers are categorized based on the underlying pathophysiological mechanisms they reflect, including markers of inflammation and oxidative stress, neuronal and endothelial injury, and those related to hemostasis and fibrinolysis. Their biological significance is evaluated through the spectrum of their diagnostic sensitivity and specificity and their potential integration into clinical practice. In addition, many of these biomarkers offer prognostic insights, helping to predict the likelihood of complications, recurrent stroke, or poor functional recovery. Furthermore, their role as a potential tool for the differential diagnosis of patients presenting with minor or nonspecific neurological symptoms and therapeutic monitoring is emphasized. Despite the promising potential of these biomarkers, their translation into routine clinical use remains limited.

## 1. Introduction

Stroke remains one of the leading causes of long-term disability and is the second most common cause of death globally [1]. This substantial global health burden underscores the critical need for rapid recognition of symptoms and timely initiation of effective treatment to optimize clinical outcomes.

A deeper understanding of the clinical pathophysiology of AIS, along with better awareness of its potential clinical manifestations, has significantly improved diagnostic timelines. This progress is largely due to the integration of advanced diagnostic modalities, which have become essential components of modern stroke assessment protocols [2,3,4].

Despite these advancements, and the growing number of well-organized stroke units across the globe, a considerable proportion of AIS cases continue to be misdiagnosed or inadequately treated during the acute phase [5].

In view of these challenges, the development of a reliable blood-based biomarker to assist in the early and accurate diagnosis of AIS holds great promise. An ideal biomarker for ischemic stroke would be rapid, non-invasive, cost-effective, and closely linked to stroke pathophysiology, while also reflecting disease severity and the patient’s response to treatment, leading to faster decision-making and more personalized patient management.

In the context of acute ischemic stroke (AIS), biomarkers play a vital role in enhancing our understanding of the underlying pathophysiological processes. These biological indicators—detectable in blood or cerebrospinal fluid—offer valuable insights into a range of mechanisms that are activated in response to an ischemic event, including inflammation, oxidative stress, blood–brain barrier (BBB) disruption, and neuronal injury or death [6].

By reflecting these complex biological responses, biomarkers contribute significantly to the early and accurate diagnosis of AIS. Moreover, they can assist in distinguishing between different stroke subtypes, which is essential for guiding appropriate therapeutic interventions [7,8]. In addition to their diagnostic applications, biomarkers are increasingly recognized for their value in monitoring disease progression, evaluating treatment effectiveness, and predicting clinical outcomes. This makes them highly useful for orchestrating patient-specific management strategies [7,8].

Compared to conventional imaging techniques, biomarker analysis offers a practical alternative. Serum biomarker testing is easier to perform, requires less specialized equipment, and can be repeated over time to allow for dynamic monitoring of a patient’s condition. These advantages position biomarkers as useful tools in both acute and long-term stroke care, enhancing the precision and responsiveness of clinical decision-making [6]. This narrative review aims to summarize the most extensively studied serum biomarkers involved in the pathophysiology of acute ischemic stroke (AIS), categorized by key biological mechanisms such as inflammation, oxidative stress, coagulation, endothelial dysfunction, and neuronal injury. It also explores their clinical relevance in early diagnosis, subtype differentiation, risk stratification, prognosis, monitoring treatment response, and guiding therapeutic decisions in AIS management. Considering the extensive number of biomarker molecules reported in the literature—both in serum and cerebrospinal fluid (CSF)—this review focuses on the most well-established and commonly studied biomarkers that are readily available to measure in most hospital settings.

## 2. Materials and Methods

This narrative review was conducted following comprehensive literature search on serum biomarkers in acute ischemic stroke, using MEDLINE (PubMed) and Google Scholar to identify studies published from January 2010 to June 2025. The predefined search strategy included the terms “ischemic stroke” combined with the variable names of commonly studied serum biomarkers in daily clinical practice.

We considered peer-reviewed publications written in English that investigated the association between serum biomarkers and AIS. Eligible study types included randomized controlled trials, observational studies, systematic and narrative reviews, and clinical practice guidelines. Studies that were thematically unrelated, conducted in animals, or categorized as editorials, commentaries, case reports, or preprints were excluded. Duplicate entries were removed prior to screening. An initial screening of titles and abstracts was independently conducted by AT and DV reviewers to identify potentially relevant articles.

Following this, a panel of experts (AS, JE, KP, EC) reviewed the full-text articles that met preliminary criteria. Studies were selected for final inclusion based on scientific rigor, clinical relevance, and clarity of presentation.

Any disagreements regarding study inclusion were resolved through consultation with OK.

## 3. Acute Ischemic Stroke Pathogenesis Pathway

Acute ischemic stroke (AIS) occurs due to the obstruction of cerebral arteries, leading to reduced cerebral blood flow, neuronal injury, and functional impairment. The most common causes include large artery atherosclerosis, cardioembolism, and small vessel disease, with less frequent etiologies such as hypercoagulable states, arterial dissection, and genetic disorders [9]. The pathophysiology involves thrombosis and cerebral ischemia, driven by platelet–fibrin interactions and abnormal activation of the coagulation cascade [10,11].

AIS progresses through several overlapping phases:

- Hyperacute Phase (0–6 h): Energy failure leads to neuronal swelling and excitotoxicity. Early reperfusion can save tissue but may worsen brain edema due to blood–brain barrier (BBB) disruption.
- Acute Phase (6 h to 3–4 days): Inflammation dominates, with reactive oxygen species and cellular debris activating immune cells, worsening BBB damage and maintaining the inflammatory cycle.
- Subacute Phase (Day 7 onward): Inflammation shifts toward repair, with anti-inflammatory responses, BBB stabilization, and angiogenesis promoting recovery.
- Chronic Phase (After 6 weeks): The BBB nearly normalizes, though low-grade inflammation persists. Recovery continues through neuroplasticity and tissue remodeling [11].

The complex pathophysiology of AIS involves numerous biological pathways and mediators, many of which can be detected in blood and used as potential biomarkers for diagnosis and prognosis. These include markers related to coagulation and fibrinolysis, endothelial dysfunction, inflammation, and neuronal or axonal injury [7].

**Figure 1:**
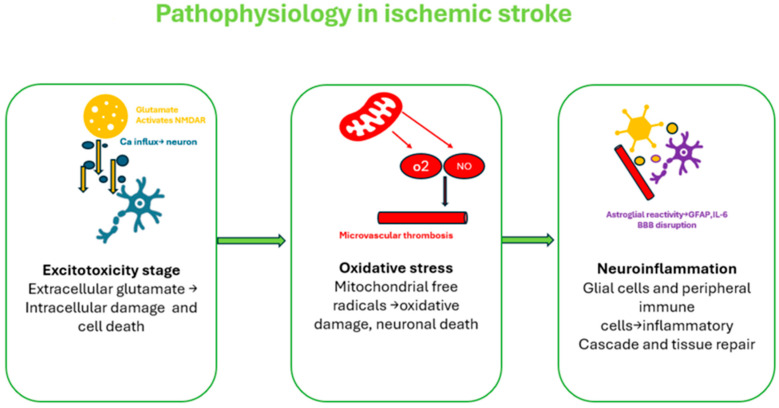
Acute Ischemic Stroke Pathophysiology. AIS initiates a cascade of ischemia-induced cellular injury, oxidative stress, inflammation, and BBB disruption [11,12,13].

During an acute ischemic stroke (AIS), three major interconnected pathophysiological processes are initiated, as illustrated in Figure 1—excitotoxicity, oxidative stress, and neuroinflammation—each contributing significantly to neuronal injury and functional impairment [12].

Excitotoxicity, an early event in ischemia, results from energy failure and impaired ATP-dependent ion transport, leading to excessive extracellular glutamate. This overstimulates NMDA and AMPA receptors, causing calcium and sodium influx, which activates damaging enzymes, disrupts mitochondrial function, and ultimately leads to neuronal death [10,12,13].

Oxidative stress rapidly follows ischemia and reperfusion, driven by excessive production of reactive oxygen and nitrogen species (ROS/RNS) from dysfunctional mitochondria and enzymes such as NADPH oxidase. These free radicals overwhelm antioxidant defenses, causing damage to lipids, proteins, and DNA, thereby contributing to neuronal death and disruption of the BBB [10,12,13].

Neuroinflammation is central to acute ischemic stroke (AIS) pathology, beginning with glial activation and immune cell infiltration. Initially, microglia and astrocytes adopt pro-inflammatory phenotypes, releasing cytokines (e.g., TNF-α, IL-1β) that exacerbate inflammation. Neutrophils, followed by monocytes and lymphocytes, further disrupt the blood–brain barrier via MMP-9, leading to edema and neuronal injury. Systemic inflammation is reflected by increased CRP, fibrinogen, and adhesion molecules. In later stages, glial cells may shift to anti-inflammatory roles, supporting repair, with GFAP indicating neuronal damage [12,13].

Together, these three core mechanisms create a harmful feedback loop that accelerates brain tissue damage, limits recovery, and complicates therapeutic interventions in AIS.

In summary, the pathogenesis of AIS is multifactorial, involving vascular occlusion, inflammation, immune responses, and cellular injury. These processes produce various bioactive molecules that not only contribute to disease progression but also serve as useful biomarkers for evaluating stroke severity, predicting outcomes, and guiding treatment decisions [13,14]. The relationship between pathophysiology and the associated biomarkers is summarized in Table 1.

## 4. AIS Serum Biomarkers: Toward a Pathophysiology-Guided Framework for Clinical Translation

### 4.1. Oxidative Stress Biomarkers

Serum uric acid (SUA) is the product of purine metabolism in humans and is primarily excreted by the kidneys. Under physiological conditions, it serves as a potent antioxidant, effectively scavenging free radicals and thereby mitigating oxidative damage to cells [15]. At the onset of AIS, SUA levels demonstrate a nonlinear relationship with the risk of poor functional outcome [16]. SUA concentrations are often markedly elevated during the acute phase of stroke and have been associated with increased mortality rates, suggesting a potential role for SUA as a prognostic biomarker for adverse outcomes, including death [32,33]. Specific SUA cut-off values have also demonstrated significant correlations with stroke severity—as measured by the National Institutes of Health Stroke Scale (NIHSS)—and with functional disability, assessed using the modified Rankin Scale (mRS) [15]. Notably, elevated SUA levels have been significantly linked to all-cause mortality, with a stronger association observed particularly among female patients, underscoring the importance of sex-specific analyses in future research [34]. However, a recent meta-analysis found no significant association between SUA levels and functional outcomes at three months post-stroke, raising questions about the consistency of these findings [35].

Collectively, these findings suggest that SUA is a dynamic biomarker depending on its concentration, making it a promising indicator for prognosis and recurrence risk in AIS.

### 4.2. Inflammatory Biomarkers

Inflammation is one of the central pathophysiological mechanisms activated during AIS. It plays a crucial role not only in the initial injury but also in the progression and secondary damage following the ischemic event [12,13]. Among the various inflammatory mediators examined in the literature, C-reactive protein (CRP) and interleukin-6 (IL-6) have emerged as particularly significant.

C-reactive protein (CRP) is a well-established acute-phase marker of inflammation, predominantly synthesized by the liver in response to systemic inflammatory stimuli [36]. CRP is effective in detecting subtle, low-grade inflammatory activity [37]. Research has demonstrated that CRP plays a key role in vascular inflammation by modulating the expression of adhesion molecules and nitric oxide within the vascular endothelium, thereby contributing to a self-perpetuating atherothrombotic process [17].

CRP has emerged as a potential etiological marker for AIS. In one study, investigators examined the relationship between CRP levels and thrombus origin, finding that cardioembolic clots contained significantly higher CRP levels compared to those associated with large artery atherosclerosis or cryptogenic sources [18]. This suggests that inflammation may play a more prominent role in clot formation in cardioembolic strokes, reinforcing earlier findings on serum CRP and further supporting its utility as a biomarker for stroke etiology. Specifically, elevated CRP levels within thrombi may point to a cardioembolic source as the potential etiological factor [18].

Among the TOAST stroke subtypes, CRP levels have been documented to be highest in patients with cardioembolic (CE) stroke, followed by those with large artery atherosclerosis (LAA), and lowest in small artery occlusion (SAA). This gradient mirrors the extent of brain tissue injury, with more extensive cerebral damage—typical of CE strokes—eliciting a stronger systemic inflammatory response and higher CRP concentrations. In contrast, the smaller, localized infarcts associated with small vessel disease trigger a milder inflammatory response and thus lower CRP levels [38,39,40,41].

Elevated CRP levels have also been inversely associated with favorable outcomes following intravenous thrombolysis (IVT), indicating reduced treatment efficacy and safety in AIS patients with exacerbated inflammatory activity [42]. Non-infectious augmentation in CRP has been identified as independent predictor of poor short- and long-term functional outcome, as well as in-hospital mortality [43]. Moreover, increased CRP levels are significantly associated with a higher risk of hemorrhagic transformation (HT) after IVT [44]. Further evidence supports a consistent association between high CRP levels and poor clinical outcomes in AIS patients, including those undergoing mechanical thrombectomy (MT) and patients with stroke subtypes such as LAA [45,46,47].

In addition, the CRP/high-density lipoprotein cholesterol (HDL-C) ratio has also emerged as a significant predictor of unfavorable long-term outcome [48]. Elevated hs-CRP levels are independently linked to increased risks of functional decline, stroke recurrence, in-hospital mortality, and poor prognosis both at discharge and during long-term follow-up [49]. Conversely, lower CRP concentrations are associated with advantageous clinical results, particularly in patients receiving guideline-adherent medical therapy for AIS [50]. The predictive value of CRP is further reinforced by its correlation with baseline clinical variables such as NIHSS score, fasting glucose, and age [51], highlighting its utility as a prognostic biomarker in AIS [52,53,54].

Low-grade systemic inflammation, as reflected by elevated CRP levels, has also been identified as an independent predictor of recurrent ischemic events, particularly in patients with minor ischemic stroke or transient ischemic attack (TIA) [55]. Notably, the combination of elevated CRP levels and imaging evidence of multiple acute infarctions significantly enhances the precision of one-year stroke risk stratification compared with either marker assessed individually, emphasizing the value of integrating inflammatory biomarkers with neuroimaging for individualized risk assessment in minor stroke or TIA [56].

Interleukin-6 (IL-6) serves as a key pro-inflammatory cytokine involved in AIS pathophysiology. Primarily produced by astrocytes, microglia, neurons, and endothelial cells within the central nervous system, IL-6 acts as a signaling molecule between immune cells, vascular endothelium, and brain tissue [19,20]. Its levels rise rapidly—within six hours—after stroke onset and it is considered as a potential biomarker for both diagnosis and prognosis not only in AIS but also in TIAs [8,19,20]. Although IL-6 may also display a protective role in later recovery phases, early elevation is typically linked to worse outcome [20].

The association between IL-6 levels and cerebrovascular events such as silent lacunar infarcts and TIAs has gained increasing attention. Studies in individuals with silent lacunar infarcts suggest that elevated IL-6 levels may serve as an early biomarker of subclinical small vessel damage [57]. Similarly, patients who experience TIAs with raised IL-6 concentration are at higher risk for subsequent strokes and tend to have a greater burden of vascular pathology [58], supporting a potential role for IL-6 in identifying individuals vulnerable to cerebrovascular events, particularly those involving small vessel disease or minor ischemic episodes [19,57,58,59,60,61].

IL-6 demonstrates limited utility as a prognostic biomarker due to its lack of specificity in distinguishing stroke from other inflammatory or systemic disorders [19,59,60]. A pivotal study further clarified the dynamics of IL-6 in AIS, revealing that while elevated IL-6 levels at stroke onset may sometimes reflect underlying or undiagnosed comorbidities, a consistent rise in IL-6 within the first 24 h post-onset—observed in approximately 90% of AIS patients—strongly support stroke as the primary trigger for this acute inflammatory response [62]. Despite variability between individuals, IL-6 followed a remarkably consistent temporal trajectory within the first 24 ± 6 h after symptom onset. Circulating IL-6 concentrations more than doubled within just 26 min of stroke onset, with a median increase of approximately 28% every two hours, underscoring its potential as a temporal biomarker for estimating stroke onset time [62].

Furthermore, IL-6 may be more strongly linked to long-term rather than immediate prognosis in AIS patients. Elevated IL-6 levels have indicated significant associations with higher NIHSS scores, worse mRS scores, and increased mortality at three months post-stroke, highlighting its potential utility as a prognostic biomarker for poor functional outcomes and long-term survival [61].

### 4.3. Thrombus Formation Biomarkers

Platelets contribute essentially to the development of atherosclerosis, thrombosis, and ischemic stroke by promoting clot formation at sites of vascular injury [20]. Thrombi can originate from the heart, particularly in atrial fibrillation, or from ruptured atherosclerotic plaques in the carotid arteries, yet determining the precise source remains challenging in clinical practice [63]. Since most ischemic strokes are result from embolic events [20,63], this uncertainty complicates treatment decisions and the implementation of effective secondary prevention strategies.

D-dimer constitutes a fibrin degradation product indicating thrombin generation and fibrinolysis. In general, it is typically low in healthy individuals but rises significantly during acute thrombotic events [20]. While elevated D-dimer levels can serve as a marker of thrombus formation and have shown stability following ischemic stroke, their clinical utility is limited by low specificity, as levels can also be influenced by inflammation, infection, malignancy, venous thromboembolism and other conditions [21,22].

Elevated D-dimer levels can aid in identifying cryptogenic ischemic stroke patients who may benefit from cancer screening [8,20,21,22], particularly when combined with markers such as CRP, high white blood cell or platelet counts [64], and multi-territory ischemic lesions. Exceptionally high D-dimer levels, especially alongside MRI findings like bihemispheric infarcts, may help detect occult cancer within a year post-stroke [65].

In AIS patients with non-valvular atrial fibrillation (NVAF) who are not anticoagulated, elevated D-dimer is associated with left atrial enlargement (LAE), reflecting a hypercoagulable state and structural atrial changes. Combining D-dimer with echocardiographic findings may improve risk stratification and guide anticoagulation strategies in NVAF-related AIS [66].

D-dimer also serves as a potential biomarker for early detection of acute aortic dissection (AAD) in patients with stroke, with isolated neurological symptoms, warranting whole-body contrast-enhanced CT when markedly elevated [67]. Additionally, elevated admission D-dimer independently predicts ischemic stroke in infective endocarditis patients, highlighting the need for close monitoring during the first three months [68].

D-dimer levels are significantly higher in CE strokes compared to other TOAST subtypes, with the lowest levels observed in SAA, reflecting the greater coagulation and fibrinolytic activity in CE strokes versus localized vessel occlusion in SAA. Early measurement of D-dimer may serve as an independent predictor of LVO, aiding prehospital triage and directing patients to specialized stroke centers [38,69,70,71,72,73,74,75]. Elevated D-dimer is also associated with potential embolic sources (PES), particularly in ESUS, where right-to-left shunts, with or without deep vein thrombosis, contribute to increased levels [76]. D-dimer levels in AIS vary with time from symptom onset, a pattern not seen in TIA, highlighting its potential as a biomarker for thrombotic risk and stroke characterization [77].

Thrombotic and inflammatory markers, including D-dimer, albumin, CRP/ALB ratio, neutrophil and lymphocyte counts, NLR, and LMR, have demonstrated prognostic value for AIS patients undergoing IVT [78,79]. Novel composite biomarkers, such as C-NLR and C-LMR, further enhance predictive accuracy [79]. D-dimer correlates with both short- and long-term outcomes after MT [80] and is associated with futile recanalization when elevated post-procedure [81]. Early assessment of D-dimer, especially within 4.5 h of symptom onset and in combination with renal function and coagulation markers, may improve risk stratification and prediction of in-hospital and functional outcomes following IVT and MT [82,83].

In AIS patients, elevated D-dimer at admission is associated with poorer short-term outcomes, including higher 30-day mortality, increased risk of lesion recurrence, and reduced functional recovery at 30 and 90 days [84,85]. Combining D-dimer with biomarkers such as NT-proBNP or CRP enhances prediction of early complications, long-term disability, and one-year mortality [86,87]. In elderly patients, D-dimer independently correlates with stroke severity and adverse outcomes, alongside age, atrial fibrillation, and prior transient ischemic attacks, underscoring its value as a prognostic biomarker and tool for risk stratification [88].

D-dimer has demonstrated strong prognostic value in recurrent ischemic stroke, particularly in embolic subtypes and strokes of undetermined origin [89]. In patients with NVAF, D-dimer, along with NT-proBNP and left atrial diameter, effectively predicts future ischemic events, with D-dimer serving as a key marker of embolic risk [90]. Moreover, persistently elevated D-dimer levels in hypercoagulable stroke patients, even under anticoagulation, identify those at high risk of recurrence, highlighting its utility as both a prognostic biomarker and a tool for monitoring treatment response [91].

Fibrinogen is a soluble plasma glycoprotein synthesized by the liver that functions as a key coagulation factor. Circulating in plasma at relatively high concentrations (2–4 mg/mL), it remains inactive until coagulation is triggered, a point at which, thrombin converts fibrinogen into fibrin, forming the structural framework of a blood clot [8]. Elevated plasma fibrinogen levels are associated with thrombotic activity and atherosclerosis, particularly in coronary, carotid, and peripheral arteries [23]. However, its specificity to stroke is limited, as increased levels can also occur in various inflammatory, infectious, and cardiovascular conditions [8,23,24]. In the context of AIS, multiple studies have shown that fibrinogen levels often rise within hours of onset and are associated with unfavorable clinical outcome [25]. Consequently, fibrinogen may have value as a prognostic marker for stroke severity, recovery, and recurrence risk [92]. Despite these associations, its utility as a diagnostic marker remains debated due to its lack of specificity [26].

Patients with ESUS and LAA exhibit elevated plasma fibrinogen levels compared to other stroke subtypes, reflecting enhanced prothrombotic and systemic inflammatory states. In LAA, high fibrinogen contributes to plaque formation, endothelial dysfunction, and thrombogenesis, increasing vascular risk, while in ESUS it may indicate underlying pro-inflammatory or pro-coagulant processes [93]. These findings underscore fibrinogen’s potential role in the pathogenesis of specific ischemic stroke subtypes; however, its predictive accuracy is lower than that of D-dimer, which is more sensitive in detecting thrombotic activity associated with stroke [94,95].

Elevated plasma fibrinogen at admission in AIS is associated with poorer short-term outcomes and may serve as a prognostic biomarker following IVT [96,97]. Dynamic changes in fibrinogen levels after alteplase administration provide insight into thrombolytic efficacy, with the fibrinogen-to-albumin ratio (FAR) offering enhanced predictive value for three-month post-IVT outcomes [98]. Fibrinogen plays a key role in thrombus formation and fibrinolysis; elevated levels increase thrombotic risk [99], while post-thrombolysis depletion correlates with hemorrhagic complications, particularly symptomatic intracranial hemorrhage (sICH) [100]. Comparative studies indicate that tenecteplase maintains more stable fibrinogen levels than alteplase, reducing bleeding risk [101,102]. Admission fibrinogen, FAR, blood glucose, and NIHSS scores are independent predictors of HT post-IVT, supporting their use in early risk stratification [103,104,105]. Concerning MT, it has been reported that lower fibrinogen levels in cases of spontaneous HT without atrial fibrillation (AF), and elevated fibrinogen levels in HT occurring post- MT, have both been associated with increased severity of HT [106].

Elevated plasma fibrinogen in AIS exhibits a positive, nonlinear association with poorer functional recovery at three months [107], indicating its potential as a prognostic marker for neurological outcomes, morbidity, and mortality [108]. The FAR further predicts unfavorable 3-month outcomes, particularly in acute pontine infarction, highlighting the role of inflammatory biomarkers in stroke prognosis [109]. High fibrinogen levels are also independently linked to cognitive decline and reduced functional independence post-stroke, underscoring their value in risk stratification for post-stroke cognitive impairment [110].

Fibrinogen is an independent prognostic marker for stroke recurrence, with its predictive value influenced by the timing of blood sampling. Elevated fibrinogen is associated with cardiovascular risk factors and promotes atherogenesis via inflammatory pathways, alongside markers such as IL-6 and CRP. Although anti-inflammatory therapies, including colchicine and canakinumab, have demonstrated efficacy in cardiovascular disease, their potential role in stroke prevention remains to be established [111].

### 4.4. Cardiac Function Biomarkers

During AIS, CNS metabolic disturbances may impair cardiac function, often reflected by elevated myocardial injury markers (cardiac troponin T-cTn) and hemodynamic stress indicators (N-terminal pro-B-type natriuretic peptide NT-proBNP [112].

Elevated serum levels of cardiac troponin T (cTnT) have been independently linked to a higher risk of death or major disability following ischemic stroke, indicating potential prognostic value [27]. In thrombolysis-treated patients, increased troponin I (cTnI) levels were associated with more severe stroke and a greater burden of comorbidities. Additionally, elevated admission cTnI levels were linked to a higher risk of 5-year mortality, suggesting their potential use in predicting long-term outcome [113].

Also, cardiac biomarkers—particularly serum troponin levels—have been found to be associated with LVO in patients with AIS [114,115]. Elevated troponin levels also correlate with the severity of AIS, as reflected by higher scores on the NIHSS [116,117]. However, elevated cTnT is frequently observed in AIS patients, even in the absence of acute coronary events [118].

cTnT levels measured upon hospital admission have been found to be significantly associated with an elevated risk of 90-day mortality in patients diagnosed with AIS who undergo treatment with IVT. This association underscores the prognostic value of cTnT as a biomarker for short-term outcome in this clinical population, suggesting that early myocardial injury or stress may contribute to adverse neurological or systemic events following reperfusion therapy [119]. In addition, in AIS patients undergoing MT, dynamic changes in cTnI—particularly those characterized by a rising trend over time rather than isolated elevation at a single time point—emerged as independent predictors of 90-day all-cause mortality. This association was especially pronounced in older individuals [120]. Taken together, these findings suggest that while static elevation of cardiac troponins at admission may signal poor prognosis following IVT, it is the dynamic pattern of troponin fluctuations—especially rising trends—that holds greater prognostic significance in the context of MT, particularly among geriatric patients.

Elevated cardiac troponin levels, particularly cTnT and cTnI, are consistently linked to adverse clinical outcomes in patients with AIS [27,121]. Evidence indicates that both static elevations at baseline and dynamic increases over time independently predict higher short- and long-term mortality, increased risk of major cardiovascular events, early neurological deterioration in patients with AF, and unfavorable discharge disposition [122,123]. Furthermore, troponin elevation correlates with worse functional outcome, as reflected by higher mRS scores and increased incidence of major disability [124]. The prognostic value of troponin is particularly notable in patients with comorbid conditions that may exacerbate myocardial injury or reflect underlying systemic stress [125]. Combining cardiac biomarkers such as troponins with electrocardiographic parameters (e.g., ET and prolonged QTc interval) has been shown to enhance risk stratification for long-term mortality [126]. Importantly, troponin elevation during the acute phase of stroke (within the first 7 days) has implications that extend beyond hospitalization, predicting long-term adverse outcomes including stroke-related, cardiac, and cancer-related mortality [127,128]. In summary, serum troponin elevation serves as a valuable prognostic biomarker in AIS, associated with increased mortality, major adverse cardiovascular and cerebrovascular events, and poor functional recovery [129,130,131,132].

High-sensitivity cardiac troponins are associated with increased cardiovascular risk in patients with ischemic stroke and TIA [133]. Elevated cTnT levels show a dose-dependent relationship with higher risk of recurrent vascular events and mortality within three years following a first mild to moderate ischemic stroke [134]. Similarly, higher cTnI concentrations are linked to a greater risk of incident stroke in the general population, regardless of stroke subtype [135].

NT-proBNP (N-terminal pro-B-type natriuretic peptide) is a biomarker released by cardiac myocytes in response to stimuli such as wall stress [20]. Increased levels are specifically associated with certain stroke subtypes, including cardioembolic stroke and newly identified atrial fibrillation [20,136]. In the context of cryptogenic stroke, elevated BNP levels have shown strong predictive value for identifying underlying atrial fibrillation (AF) [137]. Specifically, NT-proBNP independently predicted the detection of paroxysmal atrial fibrillation (PAF) in AIS when sinus rhythm was detected at the time of admission [138]. A systematic analysis further supports these findings, confirming the utility of NT-proBNP as a reliable biomarker for detecting atrial fibrillation in patients with cryptogenic stroke. NT-proBNP exhibits commendable diagnostic accuracy, ranging from good to very good, in identifying AF in individuals who have experienced cryptogenic stroke [139].

Elevated levels of NT-proBNP have been associated with larger infarct core volumes on CT perfusion (CTP) imaging in AIS [140]. Additionally, NT-proBNP serves as a significant biomarker for distinguishing AIS from hemorrhagic stroke [141].

Regarding the response to acute-phase therapies, elevated levels of NT-proBNP have been positively correlated with the occurrence of HT in patients with AIS undergoing IVT [142].

Concerning neurological deficits, elevated levels of NT-proBNP are independently associated with suboptimal functional outcome at 90 days in patients with AIS. Higher NT-proBNP concentrations significantly predict worse mRS scores at 90 days, underscoring their prognostic utility across the AIS population [140]. Additionally, it has also emerged as a valuable biomarker for predicting in-hospital mortality. Individuals with large infarcts exhibited significantly higher NT-proBNP levels compared to those with small or medium-sized infarcts. This rise in NT-proBNP may reflect infarct progression or mass effect, suggesting worsening clinical status. Therefore, serial monitoring of NT-proBNP levels may aid in early identification of patients at higher risk for adverse outcomes, allowing for targeted and timely tailored clinical interventions [143].

Upon evaluating the likelihood of stroke recurrence, increased serum levels of NT-proBNP measured within the first hours following TIA have been associated with a significantly increased risk of subsequent stroke, whether ischemic or hemorrhagic. Notably, the magnitude of this risk varied according to the etiopathogenic subtype of TIA—being highest in cardioembolism (approximately 26-fold increase), followed by TIAs of undetermined origin (five-fold increase), and atherothrombotic TIAs (nearly two-fold increase). In cases of TIA of undetermined origin, early measurement of NT-proBNP may be informative. Elevated levels should prompt a comprehensive diagnostic workup, particularly aimed at identifying a potential cardioembolic source [144].

### 4.5. Neuronal and Axonal Injury Markers

Recent advances highlight the role of axon structure, axon–glia interactions and related signaling in stroke. In AIS, BBB disruption releases biomarkers such as Glial fibrillary acidic protein (GFAP) and neurofilament light chain (NFL), reflecting astroglial and neuroaxonal damage. While these markers may aid in prognosis, clinical use is limited by variable sampling and lack of standardized cutoffs [145].

Glial fibrillary acidic protein (GFAP) is an intermediate filament protein found mainly in astrocytes and is typically absent from the bloodstream under normal conditions [146]. Emerging research supports the broader clinical potential of GFAP as a biomarker in various neuroinflammatory and neurodegenerative conditions, as well as in systemic diseases affecting the central nervous system [147]. Evidence indicates it functions as a marker of astrocytic integrity and reactive gliosis, with its expression increasing significantly in both ischemic and hemorrhagic strokes—more so in hemorrhagic events [19].

As part of the differential diagnosis, GFAP has demonstrated promising diagnostic accuracy in distinguishing ICH not only from ischemic stroke but also from stroke mimics, as its concentration is significantly elevated in patients with ICH compared to those with AIS [8,60,148]. In hemorrhagic stroke, GFAP levels rise rapidly, peaking within 2–6 h due to early BBB disruption and neuronal damage [8,19,28]. In AIS, GFAP levels increase more slowly, starting around 8 h after onset and peaking between days 2 and 5, reflecting delayed cell necrosis and BBB breakdown [8,29,149,150,151,152,153].

Higher GFAP levels have also been associated with increased stroke severity and a history of previous stroke, suggesting a link between elevated GFAP and both acute and chronic neurological/brain injury [60,154]. Importantly, point-of-care GFAP testing using blood samples collected during the prehospital phase has shown potential in identifying ICH with moderate to high positive predictive value [30]. When combined with clinical predictors, GFAP measurements could enhance early identification of ICH in the field, enabling the possibility of initiating targeted interventions during prehospital transfer to specialized stroke centers. This integrated approach could significantly improve outcome by expediting appropriate care for patients with hemorrhagic stroke [155].

In the context of AIS, serum GFAP levels effectively differentiate between patients with LVO, SVO, and healthy controls, with the highest concentrations observed in LVO cases and the lowest in controls [156]. When combined with D-dimer levels and stroke severity scales, such as the Field Assessment Stroke Triage for Emergency Destination (FAST-ED), this biomarker panel demonstrated high diagnostic performance for LVO detection with improved accuracy in targeted subgroups. Prehospital implementation of the LVO- device, which incorporates established GFAP thresholds, may facilitate rapid and accurate triage of suspected LVO patients, supporting timely transport decisions and intervention [157,158].

Compared with NFL, GFAP levels exhibited an earlier peak at Day 1 post-stroke, in contrast to NFL, which peaked at Day 7. Both GFAP and NFL concentrations demonstrated significant correlations with recovery in both longitudinal and prospective analyses. Multivariate analysis identified GFAP at Day 1 (GFAP-D1) and NFL at Day 7 (NfL-D7) as independent predictors of 3-month outcome, including NIHSS, Trunk Control Test (TCT), Functional Ambulation Categories (FAC), and Functional Independence Measure (FIM) scores [159].

Furthermore, the inclusion of GFAP significantly enhanced the predictive accuracy of the NIHSS in identifying unfavorable outcome [160]. Serum GFAP levels showed a strong positive correlation with NIHSS scores at 1-month post-stroke [161]. Higher GFAP levels corresponded with greater NIHSS scores and were also correlated with the extent of brain damage observed on CT imaging in AIS [162].

Neurofilament light chain protein (NFL) is considered as a biomarker of axonal and neuronal damage. Neurodegeneration leads to the release of cytoskeletal proteins, including neurofilaments (NFL), into CSF, and blood [163]. In AIS, serum NFL is gaining recognition for its potential in monitoring neuroaxonal damage, aiding prognosis, and guiding treatment decisions. Neuroaxonal injury significantly contributes to long-term disability and survival outcomes [164]. While NFL has been more widely studied in ischemic stroke, its role in hemorrhagic stroke remains restricted. Research has demonstrated that elevated NFL levels are correlated with both ischemic and hemorrhagic stroke [165].

Patients diagnosed with AIS due to CE or LAA were found to have the highest concentrations of NFL [60].

In terms of diagnostic characteristics of AIS, NFL concentrations, measured both acutely and at 3 months after stroke onset, have been shown to correlate significantly with infarct volume and time from stroke onset, indicating their potential as dynamic biomarkers of neuronal injury [31]. Importantly, higher NFL concentrations are associated with higher NIHSS scores mRS scores at admission [164]. In addition, NFL concentrations during the subacute phase of stroke—and the change in NFL levels between admission and the seventh day of hospitalization—have shown a strong correlation with infarct volume [166,167]. Furthermore, NFL levels appear to be elevated in AIS compared to TIA [60,168,169] and stroke mimics [169], suggesting a potential role for NFL in the differential diagnosis of acute cerebrovascular events.

In relation to clinical prognosis, NFL concentrations were predictive of functional outcomes at 3 to 6 months post-stroke, as reflected by the Barthel Index (BI) and the mRS [170,171,172]. Importantly, early neurological deterioration could be predicted as well as long-term cardiovascular risk [165,173]. In addition, NFL independently correlates with NIHSS, mRS, and Mini-Mental State Examination (MMSE) scores at the time of blood sampling, indicating its relevance across neurological, functional, and cognitive domains [165], thus it may serve as a biomarker for long-term neuropsychiatric manifestations, including cognitive decline and mental health disturbances following AIS [174]. Circulating NFL levels are also predictive of long-term cognitive changes in patients with post-stroke cognitive impairment (SCI) [175,176].

In the evaluation of stroke risk, NFL was the only neuroglial biomarker independently and significantly associated with both an increased likelihood of stroke and all-cause mortality in patients with AF who were not receiving oral anticoagulation [177]. Elevated levels appear to reflect both symptomatic and subclinical cerebral ischemic events, enhancing risk stratification in AF patients, including those without a prior history of stroke. The integration of NFL with clinical variables such as age, prior stroke history, and additional biomarkers warrants further investigation to improve individualized stroke risk prediction in this population [178].

Moreover, regarding stroke recurrence, elevated NFL concentrations have been associated with a higher risk of future stroke and are consistently increased in individuals with MRI-confirmed brain infarcts, further supporting its value as a predictive biomarker for cerebrovascular events [179]. The clinical applications of the aforementioned biomarkers in the context of AIS management are detailed in Table 2.

## 5. Discussion

This narrative review aims to highlight the clinical significance of serum biomarkers in the diagnosis of AIS, with a focus on biomolecules that are commonly measured in routine clinical practice. As the global population continues to age, the incidence of stroke is expected to rise, underscoring the importance of early diagnosis for timely therapeutic intervention. Although neuroimaging remains the cornerstone of AIS diagnosis and guides treatment decisions, such resources are often limited or unavailable in rural and resource-constrained healthcare settings. In this context, serum biomarkers represent a promising complementary tool, as they are widely accessible and can provide valuable insights into the underlying pathophysiological mechanisms of stroke.

According to the pathophysiological sequence of AIS, three major stages can be distinguished: (1) the early phase, characterized by excitotoxicity and oxidative stress; (2) the intermediate phase, involving inflammation and disruption of the blood–brain barrier (BBB); and (3) the late phase, which encompasses secondary degeneration and tissue remodeling. Systemic responses also play a key role throughout these processes. Various serum biomarkers reflect these pathophysiological changes, including markers of oxidative stress (SUA), inflammation (CRP, IL-6), glial and neuronal injury (GFAP, NFL), cardiac stress (troponin, NT-proBNP), and coagulation and fibrinolytic markers (D-dimer, fibrinogen). Additionally, emerging molecular and genetic biomarkers have shown promise, although they are not discussed in this review.

The integration of these biomarkers into clinical practice may enhance the early diagnosis of AIS and improve differential diagnosis among ischemic stroke, hemorrhagic stroke, TIA, and stroke mimics. For example, GFAP levels increase within 2–6 h in hemorrhagic stroke, whereas NFL rises acutely in ischemic stroke. When combined with neuroimaging findings and clinical scales such as the NIHSS and ASPECTS, certain biomarkers, such as NT-proBNP, troponin, and IL-6 may help predict infarct volume and stroke severity. Furthermore, biomarkers can assist in therapeutic decision-making; for instance, D-dimer, fibrinogen, troponin, NT-proBNP, and CRP levels have been associated with CE, LVO, and LAA subtypes, thus aiding in treatment decisions (IVT, M.T) and secondary prevention strategies (long-term cardiac rhythm monitoring, anticoagulation, antiplatelet therapy). In addition, biomarkers can support the monitoring of complications such as hemorrhagic transformation and post-stroke infections and may help predict both short-term outcomes and long-term disability or cognitive impairment.

Despite these promising insights, several methodological challenges and knowledge gaps persist. The absence of standardized protocols, including defined cut-off values, uniform sampling methods, and optimal timing of specimen collection—can lead to inconsistent or misleading results. Because each biomarker testing exhibits distinct kinetic patterns after stroke onset, accurate interpretation remains complex. Furthermore, comorbid conditions such as cancer, infection, or recent surgery, renal dysfunction or cardiovascular diseases can influence biomarker levels, thereby reducing their specificity and increasing the likelihood of false-positive results. Integration of biomarker data with clinical and neuroimaging findings also remains suboptimal, limiting the comprehensive assessment of stroke pathophysiology.

For these reasons, large-scale multicenter studies and the incorporation of modern analytical tools, such as multi-omics platforms, devices and machine learning, are essential to validate the diagnostic and prognostic value of serum biomarkers. Such efforts will facilitate their standardization and integration into clinical workflows, ultimately improving precision in stroke diagnosis, treatment, and outcome prediction.

## 6. Future Perspective: Microglia as a Promising Therapeutic Target

To the best of our knowledge, no single biomarker or panel of biomarkers has yet demonstrated sufficient reliability for the diagnosis and clinical management of AIS. Furthermore, emerging “omics” technologies and genetic biomarkers hold significant promise for future applications. However, their routine clinical use remains limited by high cost and technical complexity [180].

As research on this topic continues to evolve, an important focus should be the development of well-tailored therapeutic strategies. While thrombolysis and mechanical thrombectomy remain key interventions in the acute phase of stroke, a substantial number of patients are ineligible for these treatments. Within the molecular landscape of stroke, modulation of microglial function holds significant promise, particularly in attenuating the post-ischemic inflammatory response and improving neurological outcomes. However, the therapeutic potential of targeting microglia after ischemic stroke remains under investigation, as most immunoregulatory and anti-inflammatory approaches have demonstrated efficacy predominantly in animal models rather than clinical [181].

There are several clinical stages during which targeted therapies could play a pivotal role, as microglial activation follows a temporally regulated progression from early pro-inflammatory responses to later reparative states. Within the first 4 h, TLR4/NF-κB signaling is rapidly induced, marked by CD11b [182,183,184] and NF-κB p65 expression and accompanied by both pro-inflammatory mediators (IL-1β, IL-6, TNF-α, iNOS) and early anti-inflammatory signals (IL-10, CD206) [185]. LPS-induced activation at 6–24 h further reflect acute inflammatory engagement [186]. Around 3 days, caspase-1 activation indicates inflammasome involvement, while PPARγ [187] and IRF4 signaling pathways [188] promote anti-inflammatory and reparative microglial polarization, associated with CD68, CD86, and CD206 expression. Concurrently, Wnt/β-catenin signaling contributes to tissue repair [189]. During the later stages (7–35 days), PACAP [190] and STAT3/IL-13–STAT3 pathways become predominant, maintaining anti-inflammatory and neuroprotective functions, as shown by Arg1, CD206, and IBA1 expression [191,192,193].

The neuroprotective functions of microglia are attributed to their enhanced secretion of neurotrophic factors, including brain-derived neurotrophic factor (BDNF) and glial cell line-derived neurotrophic factor (GDNF), as well as anti-inflammatory cytokines such as transforming growth factor-β (TGF-β) and interleukin-10 (IL-10). Inducing microglial polarization toward M2 anti-inflammatory phenotype holds significant promise as a therapeutic approach to reduce post-stroke brain injury [194].

As stroke triggers neurochemical and vascular changes, neuroinflammatory processes overlap with neurodegeneration. This bidirectional cascade evolves into cerebral small vessel disease (CSVD)- a leading contributor to post-stroke cognitive decline-that remains an important area for future investigation. Although many biomarker associations remain correlative, emerging evidence—particularly for NfL—suggests potential causal links with disease progression, supporting the integration of biomarker profiling with neuroimaging in multimodal diagnostic frameworks for post-stroke dementia [195,196].

## 7. Conclusions

Although stroke assessment depends on neurological examination and neuroimaging, serum biomarkers provide valuable complementary information, especially when conventional findings are inconclusive. Early biomarker evaluation can support patient triage, clinical decision-making, monitoring and complication prediction. Additionally, targeting neuroinflammation represents a promising adjunctive strategy in AIS, with the potential to eliminate secondary injury and reduce long-term disability.

## Figures and Tables

**Table 1:** Biomarkers in AIS pathophysiology (↑ increased, ↓ decreased).

Pathophysiologic Category	Biomarker (Serum)	Clinical Relevance in AIS
Oxidative stress	SUA	Mildly elevated: neuroprotective role [15] Highly elevated: ↑ stroke recurrence [16]
Inflammation	CRP	↑ Vascular inflammation ↑ atherothrombotic process [17,18]
	IL-6	Rapid ↑: mediates neuroinflammation [19,20]
Thrombus formation	D-dimer	↑ in AIS (within hours) [20] ↓ specificity: associated with thromboembolic activity [21,22]
	Fibrinogen	↑ thrombotic activity [23] ↑ atherosclerosis [23,24] ↑ in AIS within hours [25] ↓ specificity [26]
Cardiac function	Troponin	↑ risk of death/major disability [27]
	NT-proBNP	↑ myocardial injury [20] ↑ wall stress [20]
Neuronal and axonal injury	GFAP	Marker of gliosis and astrocytic integrity [19]: ↑ in H.S (peak 2–6 h) [19,28], delayed elevation in AIS [29], early elevation in ICH [30].
	NFL	Early ↑ in AIS [31]

**Table 2:** Serum biomarkers in AIS: clinical utility (↑ increased).

Biomarker	Relation to Etiology/Subtype	Relation to Diagnostic Characteristics	Relation to Differential Diagnosis	Relation to Response to Therapy	Relation to H.T.	Relation to Recurrent Risk	Relation to Outcome/Prognosis
SUA	No evidence	No evidence	No evidence	No evidence	No evidence	No evidence	High levels: ↑ NIHSS ↑ mRS [15]
CRP	High levels in CE > LAA > SAA [18,38,39,40,41]	No evidence	No evidence	High levels: poor post-IVT [42], post-MT outcome [45,46,47]	High levels: ↑ risk of H.T. [44]	High levels: ↑ risk of TIA/minor stroke [55]	High levels: mortality deterioration [49]
IL-6	High levels: TIA [58], lacunar stroke [57]	High levels: infarct size ↑ NIHSS [59]	Early elevation in AIS: ~24 h ± 6 from onset; rate 28% every 2 h [62]	No evidence	No evidence	No evidence	High levels: ↑ mortality ↑ mRS [61]
D-dimer	Elevated in CE > ESUS, LAA > SAA [38,69,70,71,72,73,74,75] correlation with LVO [75] frequently elevated in cancer [64,65], LAE [66], NVAF [66], AAD [67], RLS [76], IE [65].	No evidence	TIA [77]	High levels: poor post-IVT, post-MT outcome [78,79,80]	No evidence	↑ embolic risk [89,90]	High levels: poor clinical outcome [84,85]
Fibrinogen	High levels: LAA, ESUS [93]	No evidence	No evidence	↑ FAR post-IVT alteplase: poor outcome [96,97,98]	Lower levels after IVT: ↑H.T. [100]	↑ stroke recurrence [111]	High levels: cognitive decline [110]
Troponin	High levels: LVO [114,115]	Relation to ↑ NIHSS [116,117]	No evidence	Elevation post-IVT: ↑ mortality [119]	No evidence	TIA, ischemic stroke [133].	High levels: cardiovascular events, unfavorable discharge [122,123].
NT-proBNP	High levels: CE [20,136], PAF [138], AF [137,139]	High levels: large infarct volume [140].	No evidence	No evidence	Elevation post-IVT: ↑ risk of H.T. [142]	recurrent stroke with NVAF [144]	High levels: ↑ mRS [140], infarct mass effect [143]
GFAP	High levels in LVO > SVO [156]	No evidence	Early elevation in ICH [19,28] delayed elevation in AIS [29]	No evidence	No evidence	No evidence	High levels: ↑ brain damage [60,154]
NFL	High levels in CE, LAA [60]	Positive correlation with infarct volume [16,17,18,19,20,21,22,23,24,25,26,27,28,29,30,31,32,33,34,35,36,37,38,39,40,41,42,43,44,45,46,47,48,49,50,51,52,53,54,55,56,57,58,59,60,61,62,63,64,65,66,67,68,69,70,71,72,73,74,75,76,77,78,79,80,81,82,83,84,85,86,87,88,89,90,91,92,93,94,95,96,97,98,99,100,101,102,103,104,105,106,107,108,109,110,111,112,113,114,115,116,117,118,119,120,121,122,123,124,125,126,127,128,129,130,131,132,133,134,135,136,137,138,139,140,141,142,143,144,145,146,147,148,149,150,151,152,153,154,155,156,157,158,159,160,161,162,163,164,165,166,167], ↑ NIHSS [164]; strong association with time onset [31].	Elevated in AIS > TIA [49,169,170], mimics [169]	No evidence	No evidence	subclinical events, related with AIS in AF [179].	High levels: ↑ mRS [170,171,172], BI [170] post-stroke cognitive impairment [175,176].

## Data Availability

The datasets used and/or analyzed during the current study and Supplementary Materials are available from the corresponding author upon reasonable request.

## Supplementary Materials

The following supporting information can be downloaded at: https://www.mdpi.com/article/10.3390/jcm14217748/s1, Table S1: PRISMA GUIDELINES CHECKLIST.

## Abbreviations

AIS: Acute Ischemic Stroke; CRP: C-Reactive Protein; GFAP: Glial fibrillary acidic protein; H.S: Hemorrhagic Stroke; IL-6: Interleukin-6; NFL: Neurofilament light chain; SUA: Serum Uric Acid. AAD: Acute Aortic Dissection, AF: Atrial Fibrillation, BI: Barthel Index, CE: Cardioembolic stoke CRP:C-Reactive Protein, ESUS: Embolic Stroke of Undetermined Source, FAR: Fibrinogen to Albumin Ratio, GFAP: Glial fibrillary acid protein, H.T.: Hemorrhagic transformation, ICH: Intracerebral Hemorrhage, IE: Infective Endocarditis, IVT: Intravenous thrombolysis, LAA: Large Artery Atherosclerosis, LAE: Left Atrial Enlargement, LVO: Large vessel occlusion, MMSE: Mini -Mental State Examination, M.T: Mechanical Thrombectomy, NIHSS: National Institutes of Health Stroke Scale, NVAF: Non Valvular Atrial Fibrillation, mRS : Modified Rankin Scale, PAF :Paroxysmal Atrial Fibrillation, RLS: Right to Left shunt, SAA: Small Artery Occlusion, SVO: Small Vessel Occlusion, TIA: Transient Ischemic Attack.
