# Supplementary material for: Serum Biomarkers in Acute Ischemic Stroke: Clinical Applications and Emerging Insights
Source: J Clin Med. 2025 Oct 31;14(21):7748. doi: 10.3390/jcm14217748 (PMC12610523; doi:10.3390/jcm14217748)
Supplement: Supplementary file 1 [file jcm-14-07748-s001.zip › jcm-3900594-supplementary.pdf]

TableS1: PRISMA GUIDELINES CHECKLIST

**Section**	**How Addressed in this Narrative Review**
Title	Serum Biomarkers in Acute Ischemic Stroke: Clinical Applications and Emerging Insights
Abstract	Abstract non-structured into Background, Scope, Key Findings, and Conclusions.
Rationale	Explain the importance of measuring serum biomarkers in acute ischemic stroke (AIS) and their relevance for integration into routine clinical practice
Objectives	The aim is to summarize recent advances in the most widely studied biomarkers of acute ischemic stroke
Eligibility criteria	Not a systematic search: In this narrative review we focused on recent studies (2010-2025) considered most relevant by the authors
Information sources	Main databases/Journals: MEDLINE (PubMed) and Google Scholar
Search strategy	We used the term "ischemic stroke" combined with the variable names of commonly studied serum biomarkers in daily clinical practice
Study selection	We included: Key peer-reviewed English-language articles, including pivotal trials and high-impact reviews.
Data collection and items	Narrative review: no extraction tables
Risk of bias	Not systematically assessed
Synthesis methods	Description of biomarkers according to AIS pathophysiology and clinical application
Results	Organized in five sections:1. oxidative stress biomarkers, 2. Inflammatory biomarkers,3. Thrombus Formation biomarkers, 4. Cardiac function biomarkers, 5. Neuronal and axonal injury biomarkers
Limitations	As this is not a systematic review, some relevant studies may have been missed

Conclusions	Serum biomarkers in AIS are increasingly useful tool to assist diagnosis and treatment and predict outcome. However, further validation is required in diverse populations
Other information	Registration: N/A. Funding: N/A, conflicts of interest: N/A
